# Effects of Model, Method of Collection, and Topography on Chemical Elements and Metals in the Aerosol of Tank-Style Electronic Cigarettes

**DOI:** 10.1038/s41598-019-50441-4

**Published:** 2019-09-27

**Authors:** Monique Williams, Jun Li, Prue Talbot

**Affiliations:** 1 Department of Molecular, Cell, and Systems Biology, University of California, Riverside, Riverside, California USA; 2 Department of Statistics, University of California, Riverside, Riverside, California USA

**Keywords:** Metals, Medical research, Mass spectrometry

## Abstract

Our purpose was to examine the effect of model, puffing topography (voltage, air-flow, puff interval), and method of collection on 19 elements/metals in aerosols from six tank-style electronic cigarettes (EC). Aerosols were collected from six brands using a cold trap or impinger and various puffing topographies. 19 elements were quantified using inductively coupled plasma optical emission spectroscopy. 16 elements/metals were present and quantified in the aerosols. The total concentrations of elements/metals ranged from 43 to 3,138 µg/L with the impinger method of collection and 226 to 6,767 µg/L with the cold trap method. The concentrations of individual elements were often similar across brands and across topographies. Some elements (e.g., zinc) were present in most aerosols, while others (e.g., cadmium, titanium, vanadium) were rarely found. Concentrations of some elements (e.g., lead) increased in aerosols as voltage/power increased. The model with fewest metal parts in the atomizer had the fewest metals in its aerosols. Most elements/metals in the aerosols have been found previously in the atomizers of EC. All tank-style aerosols had elements/metals that appeared to originate in the atomizers, and concentrations increased with increasing power. Concentrations of some elements were high enough to be a health concern.

## Introduction

Electronic cigarettes (ECs) are popular tobacco products consisting of a battery, atomizing unit, and refill fluid^1,2^. Since their introduction in 2005, the EC market has evolved, and new styles of ECs have frequently been released^3–5^. Currently, there are three basic types of ECs available. The cartomizer-style combines the cartridge and atomizing unit, which can be purchased separately from the battery^6^. In 2013, manufacturers introduced the disposable style which combined the cartomizer and the battery into a single unit, which is discarded after use^7^. Because both the cartomizer and disposable ECs resemble conventional cigarettes, they are often referred to as “cig-a-likes” or first generation products^5,8,9^.

In 2003, EC manufacturers introduced ECs with more powerful batteries and larger capacity reservoirs to store more refill fluid^4,5,10^. The larger batteries came in three forms: variable voltage, box mod, and variable wattage^11^. The new atomizers also had different designs: clearomizers (second generation), various shaped atomizers, and sub-ohm and replaceable dripping atomizers (RDA) (all mods or third generation), and all reservoirs stored larger amounts of refill fluid than their cig-a-like predecesors^4,12^. These advanced styles provided more customizability and functionality for consumers. However, the higher power batteries used in the newer EC models could alter the properties of the refill fluids and/or the elements/metals making up the atomizer. For this study, all clearomizers and mods will be referred to as “tank-style” EC.

The refill fluids that are used in tanks come in a wide range of flavors including classic tobacco, minty, fruity or sweet, and savory^3,13,14^. Refill fluids contain solvents (propylene glycol and glycerin) and flavor chemicals, such as cinnamaldehyde, vanillin, benzaldehyde, and ethyl maltol, which are cytotoxic at the concentrations used in some products^15–23^. The use of batteries with more power causes atomizing units to heat to temperatures greater than 300 °C, which can produce harmful by-products (e.g., formaldehyde and diacetyl) from the solvents and flavor chemicals in the fluid^19,24,25^.

In addition to solvents and flavor chemicals, some refill fluids contain elements/metals, such as tin, lead, copper, chromium, nickel, iron, and zinc, that also appear in EC aerosols^26^. EC themselves are made of metal components that include chromium, nickel, tin, copper and zinc, and these elements/metals have been reported in EC aerosols^26–30^. Cartomizer and disposable EC aerosols contained 22 chemical elements, mostly metals, with tin, copper, zinc, lead, nickel, and silicon having higher concentrations in EC aerosols than in conventional cigarette smoke^8,28–31^. Elements/metals, such as chromium, nickel, iron, and aluminum, have been recently reported in aerosols from tank-style EC^26^. The concentrations present in the tank-style EC aerosols were considerably higher than those in the cig-a-like counterparts^26,30^. Most aerosol elements probably come from different components of the atomizing unit, such as the nichrome wire, tin solder joints, brass clamps, insulating sheaths, and wicks^28–30^. The composition of EC aerosols and the presence of heavy metals, including some known carcinogens, is a concern due to their potential to cause adverse health effects with prolonged exposure^32–34^.

We have concentrated in this study on the element/metal emissions from six tank-style ECs using controlled laboratory conditions. The specific purposes of this study were to: (1) determine how the method of collection affects elements/metals in EC aerosols, a point not addressed in prior studies, (2) identify and quantify the elements that transfer to the aerosols produced by tank-style ECs, and (3) evaluate the effect of puffing topography (voltage, air-flow, puff interval) and battery power on elements/metals in EC aerosols.

## Materials and Methods

### Battery, tank, and refill fluid selection

For this generational study, five batteries, four tanks, and two RDA were selected based on their popularity over the past 3 years. Popularity was established by speaking with clerks at a local vape shop near the University of California Riverside (UCR) campus and mining information on leading refill fluid manufacturers’ websites. At the time of purchase all batteries and tanks were considered the most popular item in their class (Table 1). The following EC batteries were used: Ego C-Twist (Joyetech Co, ShenZhen, China), iTaste MVP 2.0 (Innokin, Henzhen, China), Nemesis (Shenzhen HCIGAR Technology Co., Ltd., Baoan District, China), iPV6X (Pioneer4you, Shenzhen iPV Vaping Technology Co, Shenzhen, Guangdong, China), and Smok Alien (Shenzhen IVPS Technology Co., Ltd, Shenzhen, China). The following tanks and RDA were used: Kangertech Protank (Kangertech, ShenZhen, China), Aspire Nautilus tank (Aspire, ShenZhen, China), Kanger T3S tank (Kangertech, ShenZhen, China), Tsunami 2.4 (Tsunami Vapor Glass, Troy, MI), Smok tank (Shenzhen IVPS Technology Co., Ltd., Shenzhen, China), and Clone RDA. Products were inventoried and stored at room temperature.

**Table 1:** Product information and topographic parameters used in the study.

Battery & Tank Combinations	Type	Year	Collection Method	Puffing Interval	Aerosol Parameters	Voltage (V)	Air flow Rate (mL/s)	Puff Volume (mL)
Ego C-Twist + Kangertech Protank 2^nd^ Generation - Clearomizer	Tank	2014	Cold Trap^a^	Continuous	LV:LA^c^	3.8 ± 0	4 ± 0^d^	17.2 ± 0
			Cold Trap	Continuous	LV:HA^c^	3.8 ± 0	19 ± 0	81.7 ± 0
			Cold Trap	Continuous	HV:LA^c^	4.8 ± 0	7 ± 0	30.1 ± 0
			Cold Trap	Continuous	HV:HA^c^	4.8 ± 0	19 ± 0	81.7 ± 0
			Impinger^b^	Continuous	HV:LA	4.8 ± 0	7 ± 0	30.1 ± 0
			Impinger	Interval	HV:LA	4.8 ± 0	7 ± 0	30.1 ± 0
Ego C-Twist + Aspire Nautilus Hybrid 2^nd^/3^rd^ Generation – Clearomizer/Mod	Tank	2014	Cold Trap	Continuous	LV:LA	3.8 ± 0	4 ± 0^d^	17.2 ± 0
			Cold Trap	Continuous	LV:HA	3.8 ± 0	19 ± 0	81.7 ± 0
			Cold Trap	Continuous	HV:LA	4.8 ± 0	7 ± 0	30.1 ± 0
			Cold Trap	Continuous	HV:HA	4.8 ± 0	19 ± 0	81.7 ± 0
			Impinger	Continuous	HV:LA	4.8 ± 0	7 ± 0	30.1 ± 0
			Impinger	Interval	HV:LA	4.8 ± 0	7 ± 0	30.1 ± 0
iTaste MVP 2.0 + Kanger T3S Hybrid 2^nd^/3^rd^ Generation – Clearomizer/Mod	Tank	2014	Cold Trap	Continuous	LV:LA	3.8 ± 0	7 ± 0	30.1 ± 0
			Cold Trap	Continuous	LV:HA	3.8 ± 0	19 ± 0	81.7 ± 0
			Cold Trap	Continuous	HV:LA	5 ± 0	7 ± 0	30.1 ± 0
			Cold Trap	Continuous	HV:HA	5 ± 0	19 ± 0	81.7 ± 0
			Impinger	Continuous	HV:LA	5 ± 0	7 ± 0	30.1 ± 0
			Impinger	Interval	HV:LA	5 ± 0	7 ± 0	30.1 ± 0
Nemesis + Clone 3^rd^ Generation – Mod/RDA	RDA^c^	2014	Cold Trap	Continuous	LV:LA	3.7 ± 0	15 ± 0^e^	64.5 ± 0
			Cold Trap	Continuous	LV:HA	3.7 ± 0	19 ± 0	81.7 ± 0
			Impinger	Continuous	LV:LA	3.7 ± 0	7 ± 0	30.1 ± 0
			Impinger	Interval	LV:LA	3.7 ± 0	7 ± 0	30.1 ± 0
Smok Alien + Smok 3^rd^ Generation – Mod/Sub-ohm	Tank	2017	Impinger	Continuous	HV:LA	5 ± 0	7 ± 0	30.1 ± 0
			Impinger	Interval	HV:LA	5.1 ± 0	7 ± 0	30.1 ± 0
iPV6X + Tsunami 2.4 3^rd^ Generation – Mod/Sub-ohm	RDA	2017	Impinger	Continuous	HV:LA	2.8 ± 0.2	7 ± 0	30.1 ± 0
			Impinger	Interval	HV:LA	3.6 ± 0.4	7 ± 0	30.1 ± 0

^a^Cold Trap Room Air was made using 15 mL/s.

^b^Impinger Room Air was made using 7 mL/s, with a United Filtration Systems, Inc (Sterling Heights, MI) Disposable In-Line Filter DI-BN50.

^c^Abbreviations: LV: LA, Low Voltage-Low Air flow rate, LV: HA, Low Voltage-High Air flow rate, HV: LA, High Voltage-Low Air flow rate, HV: HA, High Voltage-High Air flow rate, RDA, Rebuildable Dripping Atomizers.

^d^The low air flow rate was used for the LV:LA, but when the experiment was repeated with High voltage the 4 mL/s air flow rate did not work.

^e^For the LV:LA experiment, the lowest air flow rate to produce aerosol was 15 mL/s, for the HV experiments a new internal battery was used.

All experiments were performed using popular refill fluids that were purchased from a local vape shop near the UCR campus: WTF (12 mg nicotine/ml) (2014) (OMG, Los Angeles, CA), Pink Starburst (6 mg nicotine/ml) (2014) (e-Liq Cube, Norwalk, CA), and Breezy Shake (6 mg nicotine/ml) (2017) (Milkshake Liquids, City of Industry, CA). All fluids were stored at room temperature.

### Evaluation of leaching of elements/metals from glassware

To evaluate leaching from the 500-mL round bottom flasks (Pyrex, Fisher Scientific, Hampton, NH) that were used in the cold trap method of aerosol collection, new flasks were filled with 10% nitric acid/3% hydrochloric acid and allowed to soak for 24 hours. After the soaking period, a sample of the fluid was collected and analyzed for elemental content using the inductively coupled plasma optical emission spectroscopy (ICP-OES). This experiment was then repeated again. These are referred to as 1 day and 2 day samples in the Results.

Similar evaluations were done with new impingers that were then used with the impinger method of aerosol collection. The glass impingers (Kimble-Chase, Vineland, NJ) were filled with 130 mL of 2% nitric acid for 24 hours after which samples of the fluid were collected for elemental analysis using ICP-OES. This procedure was repeated for 5 days, and samples were collected and analyzed each day. In all subsequent aerosol collections done with impingers, presoaking was done for 5 days.

### ICP-OES aerosol sample preparation cold trap method

All aerosols were generated using a smoking machine as described before^28–30^. EC were puffed into a 500 mL round bottom flask covered with Parafilm and submerged in an ice bath. A small glass capillary served as an exhaust. Aerosol solutions were prepared using one atomizing unit. For each sample, 60 total puffs (4.3 seconds each) were taken, and aerosol was allowed to fully dissolve in a solution of 10% nitric acid, 3% hydrochloric acid, and 87% deionized water before the next puff was added to the flask. Room air was prepared in a similar fashion with no EC being puffed. All samples were stored in 15 mL conical vials (Falcon, Fisher Scientific, Hampton, NH). ICP-OES analysis was used to quantify the concentrations of 19 elements (aluminum, boron, cadmium, calcium, chromium, cobalt, copper, iron, lead, magnesium, nickel, potassium, silicon, silver, sodium, tin, titanium, vanadium, zinc)(Supplemental Table 1) in the aerosol using an Optima 7300 DV (Perkin-Elmer, Waltham, MA)^29,30^. ICP-OES running conditions and quality controls are described in detail in the Supplemental Materials.

### Effect of puffing topography on elemental concentrations using the cold trap method

To evaluate the effect of topography, aerosols were generated using four puffing topographies: low voltage-low air flow rate, low voltage-high air flow rate, high voltage-low air flow rate, and high voltage-high air flow rate (Table 1). Aerosols were generated as described above using each puffing parameter.

### ICP-OES aerosol sample preparation using the impinger method

Aerosols were collected at room temperature using two glass impingers set up in tandem. All impingers were soaked in 2% nitric acid for 5 days before use, and the solution was changed daily. All batteries were set to the highest voltage (2.8–5.0 V) (Table 1), and a 4.3 second puff was taken every minute using a 7 mL/s (30.1 mL puff) air flow rate (Table 1). Aerosols were bubbled through impingers into a 2% nitric acid, 98% deionized water. Room air samples were generated in a similar fashion with the air pulled through a disposable inline filter (Ultra Filtration System, Sterling Heights, MI). Three separate batches of aerosol were prepared for each brand. All samples were stored in 15 mL conical vials that had been pre-sealed with nitric acid to prevent leaching. Metals were analyzed using an ICP-OES as described above.

### Effect of puffing topography on elemental concentrations using impinger method

To evaluate the effect of topography, two puffing protocols were used. In the first, referred to as “continuous puffing”, aerosols were generated using the method as described above where a 4.3 s puff was taken every minute without breaks. In the second protocol, referred to as the “interval puffing”, aerosols were generated by taking a 4.3 s puff every minute for 10 minutes, then waiting 5–20 minutes to allow the tank and battery to cool before collecting another 10 puffs. While puff duration has varied in different reports^35–37^, we chose to use 4.3 seconds as it is likely representative of second and third generation ECs^38–40^.

### Statistical analysis of the concentrations of elements/metals found in tank-style EC aerosols

The raw data were used to statistically analyze the total and individual concentrations of the elements/metals. For total concentrations, a two-way analysis of variance (ANOVA) with the Fisher post-hoc test and Bonferroni correction was performed for each brand, with the exception of Clone, using Minitab (Minitab, Inc., State College, PA). For Clone and elements/metals with concentrations from only two topographies, an unpaired t-test with Welch’s correction was used to determine statistically significant differences between the two concentrations using Prism (GraphPad, La Jolla, CA). For any elements/metals that had concentrations from three or more topographies, a one-way ANOVA with Fisher post-hoc test and Bonferroni correction was performed using Minitab. When p values were less than 0.05, the comparison was considered significantly different. When a concentration was below the limit of detection, it was treated as a zero for the statistical analysis.

## Results

### Evaluation of elements/metals leached from round bottom flasks

**Figure 1:**
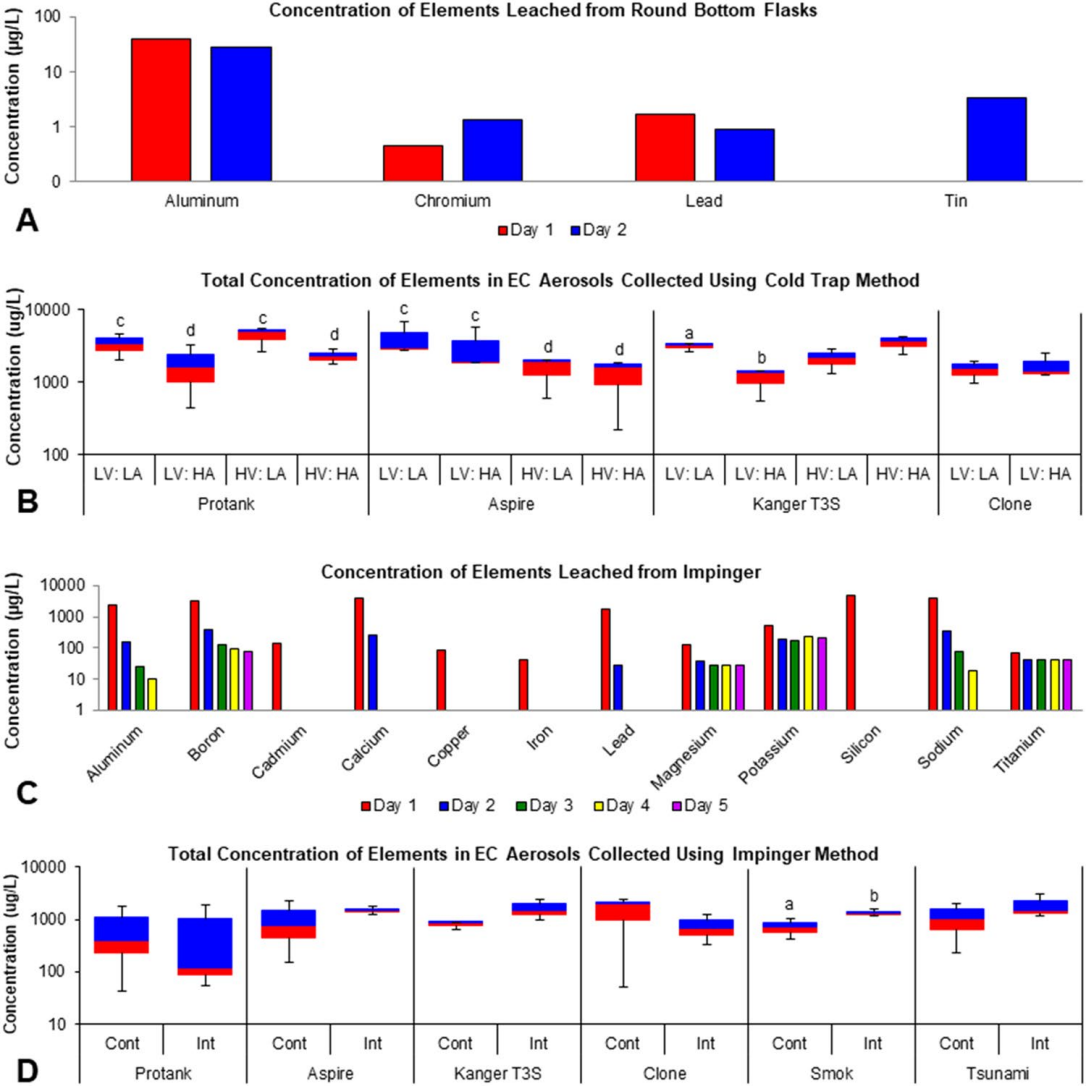
Concentration of elements/metals leached from glassware and total concentration of elements in tank-style EC aerosols across collection methods and topographic puffing parameters. (**A**) Elements/metals leached from glass round bottom flasks following 1 or 2 days of soaking in acid. (**B**) Total concentration of elements/metals present in aerosol of four EC across four puffing topographies after subtracting background levels of aluminum, chromium, lead, and tin. (**C**) Elements/metals leached from glass impingers following five 24 hour soaking periods in nitric acid. (**D**) Total concentration of elements/metals present in the aerosol of six brands of EC across two puffing topographies after subtracting background levels of boron, magnesium, potassium, and titanium. All concentrations in (**B,D**) are based on three experiments. Abbreviations: LV (Low Voltage), HV (High Voltage), LA (Low Air-Flow Rate), HA (High Air-Flow Rate, Cont (Continuous), Int (Interval). Data samples with an “a” are significantly different from samples with “b” (p < 0.05), “c” are significantly different than “d” (p < 0.01).

To determine if any of the elements/metals being studied leached from the glassware used for aerosol collection, round bottom flasks were filled with 10% nitric acid/3% hydrochloric acid for 24 hours (day 1) after which the acid was analyzed for 22 elements and the procedure was repeated (day 2) (Fig. 1A). Following 1 day of soaking, only four elements (aluminum, chromium, lead, tin) were detected in the acid, and their concentrations were low, ranging from 0.451 to 38.780 µg/L (Fig. 1A). These elements were not present in the acid prior to soaking in the flasks. In acid taken from the day 2 samples, aluminum and lead concentrations decreased slightly, while chromium and tin increased. Because these elements were presumed to be leaching from the round bottom flasks, all subsequent elemental analyses were done using flasks that had presoaked in acid for 24 hours, and the amount of aluminum, chromium, lead and tin present on day 2 (Fig. 1A) was subtracted from values measured in EC aerosols collected using the cold/trap/round bottom flask method.

### Quantification of total elements/metals in aerosols produced using the cold trap/round-bottom flask method

Aerosols were generated using four topographies that varied voltage and air flow rate. The total concentration of the 12 elements/metals in EC aerosols collected via the cold trap/round-bottom flask method are presented in Fig. 1B. For Protank, the total concentration from four topographies ranged from 442 to 5,444 µg/L, and the concentrations measured at the low air flow rate were significantly higher than concentrations measured using the high air flow rate independent of voltage (Fig. 1B, Supplemental Table 2). The total concentrations measured in the Aspire aerosol ranged from 226 to 6,767 µg/L with aerosols generated at low voltages having significantly higher concentrations than those produced at high voltages (Fig. 1B, Supplemental Table 3). For the Kanger T3S, the total concentrations of elements/metals ranged from 563 to 4,344 µg/L (Fig. 1B, Supplemental Table 4), and in the low voltage group, the concentration was significantly higher when aerosols were produced at the low air flow rate. The Clone RDA, which did not have a variable voltage and therefore was used at only one voltage, produced total element concentrations ranging from 956 to 2,496 µg/L (Fig. 1B, Supplemental Table 5). Air flow rate did not significantly alter the total element/metal concentration for the Clone RDA.

### Evaluation of elements/metals leached from impingers

To correct for elemental leaching using the impinger method of aerosol collection, all impingers were soaked in 2% nitric acid solution for 24 hours and solutions were changed every day for 5 days after which the element concentrations in the acid were quantified (Fig. 1C). Twelve elements leached into the acid solution following 1 day of soaking and were present in concentrations ranging from 507 to 4,886 µg/L (Fig. 1C). During the following 4 days of soaking, the concentration of 8 elements/metals (aluminum, cadmium, calcium, copper, iron, lead, silicon, sodium) declined until they were either no longer present or they were present in trace amounts (aluminum, boron, magnesium, titanium). Potassium concentrations dropped after 1 day of soaking, but remained about 204 ± 28 µg/L over the next 4 days (Fig. 1C). Based on these results, the concentrations of boron, magnesium, potassium, and titanium on day 5 were subtracted from the total concentrations in the EC aerosols produced using the impinger method.

### Quantification of total elements in aerosols produced using the impingers

Aerosols from all brands of ECs were generated using high voltage (2.8–5 V), low air flow rate (7 mL/s), and puffing protocols that were either continuous (one puff every minute) or done at intervals (one puff each minute for 10 minutes, followed by a 5 to 20 minutes break before the next 10 puffs) (Table 1). The total concentrations of elements/metals measured in EC aerosols prepared using the impinger method are presented in Fig. 1D. Total concentrations varied between brands and ranged from 43 to 2,392 µg/L for continuous puffing and 55 to 3,137 µg/L for interval puffing (Fig. 1D, Supplemental Tables 2–7). The large deviations in some groups in Fig. 1D further indicate variations within brands. With the exception of the Protank and Clone, concentrations were higher in the interval puffing group than in the continuous group, with significance reached for the Smok product (Fig. 1D, Supplemental Tables 2–7).

### Total concentration of elements in all brands of EC aerosol compared by method of collection and topography

**Figure 2:**
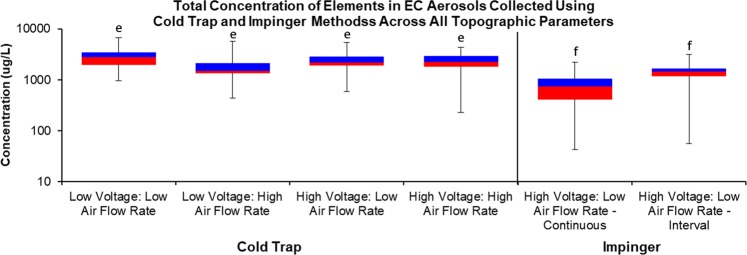
Total concentration of elements compared by method of collection and topography. The overall total concentration of all elements/metals in the aerosol of tank-style EC across both collection methods and topographic puffing parameters. All concentrations are based on three independent experiments. Brands included in these data for Cold Trap Low Voltage (Protank, Aspire, Kanger T3S, Clone), High Voltage (Protank, Aspire, Kanger T3S) and Impinger: High Voltage (Protank, Aspire, Kanger T3S, Smok, Tsunami). The bars with “e” and “f” above them are significantly different from each other (p < 0.001).

The total concentration of elements/metals in aerosols from all EC brands collected with the cold trap and impinger methods using various topographies are summarized in Fig. 2. Each box whisker represents the total concentration of elements in each topographic parameter. The total concentration for samples collected using the cold trap method ranged from 226 to 6,767 µg/L, while the samples generated using the impinger method ranged from 43 to 3,137 µg/L (Fig. 2). Concentrations of the cold trap samples were significantly higher than the samples collected using the impinger method (Fig. 2).

### Average total concentrations of individual elements/metals in EC aerosol: comparison of collection methods and topography

**Figure 3:**
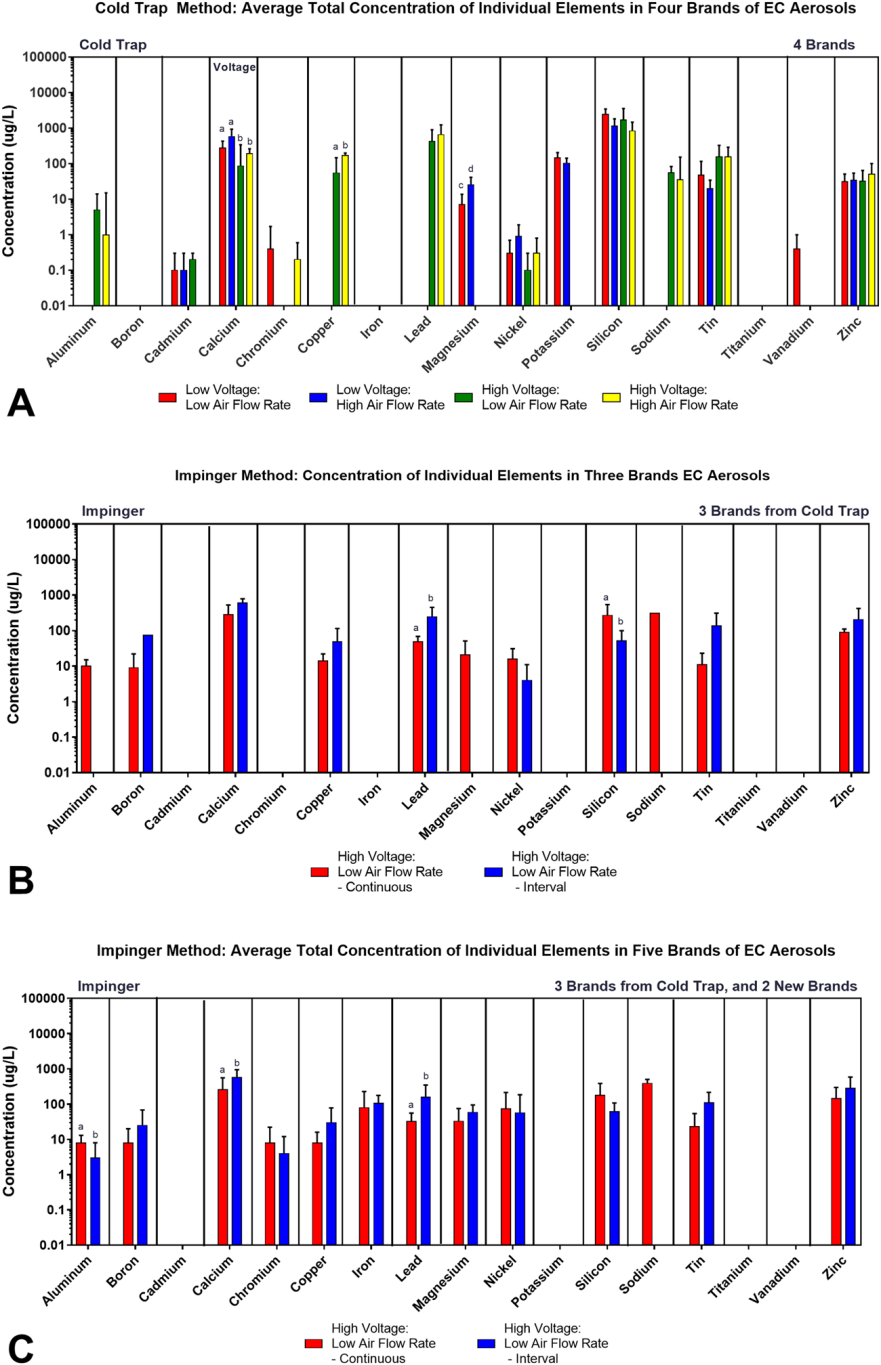
Comparison of the average total concentration of individual elements/metals in the aerosol collected using two different collection methods and different topographic parameters. (**A**) Aerosols from four brands of tank-style EC (Protank, Aspire, Kanger T3S, and low voltage for Clone) using the cold trap method. (**B**) Aerosols collected using the impinger method for the three brands of tank-style EC in Figure **A** (Protank, Aspire, Kanger T3S). (**C**) Aerosols from five brands of tank-style EC (Protank, Aspire, Kanger T3S, Smok, Tsunami) using the impinger method. All concentrations are based on three independent experiments. Data samples with an “a” are significantly different from samples with “b” (p < 0.05), “c” are significantly different than “d” (p < 0.01), samples with an “e” are significantly different from samples with an “f” (p < 0.001).

The average total concentration of each element/metal in cold trap aerosols made from Protank, Aspire, Kanger, and Clone products is shown for each topographic parameter in Fig. 3A. Boron, iron and titanium were not found in any samples collected with the cold trap. Calcium, nickel, silicon, tin, and zinc were present in aerosols collected with all four topographic parameters (Fig. 3A). Some elements appeared only when voltage was high (aluminum, copper, lead, and sodium). For calcium, the concentration was significantly higher in aerosols made using low voltage (Fig. 3A). For copper, the concentration was significantly higher in the samples made using high voltage and high air flow rate (Fig. 3A). The concentration of magnesium was significantly higher in samples prepared using low voltage and high air flow rate (Fig. 3A). These differences for calcium, copper and magnesium, while significant, were relatively small.

The total concentrations of individual elements were then examined for three brands (Protank, Aspire, and Kanger) using the impinger method to collect aerosols (Fig. 3B). This figure can be directly compared to Fig. 3A. Cadmium, chromium, potassium, titanium, and vanadium were not detected with either continuous or interval puffing. Unlike the cold trap method, boron and iron were detected with the impinger method. Aluminum and sodium were only detected in samples made using the continuous puffing protocol. The concentration of lead in the interval puffing (245 ± 208 µg/L) was significantly higher than the concentration in continuous puffing (48 ± 26 µg/L) (Fig. 3B). Silicon concentration in the continuous puffing protocol (270 ± 248 µg/L) was significantly higher than the interval method (77 ± 33 µg/L).

Figure 3C is similar to Fig. 3B except all brands are now included, i.e. those in Fig. 3B plus Smok and Tsunami. The data are similar to those in Fig. 3B, except that chromium is now present, perhaps due to higher temperatures with the sub-ohm products (Smok and Tsunami), and strikingly there was an increase in average total concentrations for some elements, such as iron, nickel, and tin, suggesting the components of these newer models could deliver more metals to the consumer. Nickel concentrations measured in the impinger samples were 10 times higher than those measured in the cold trap samples (Fig. 3A,B). Cobalt, silver, and titanium were the only three elements that were not detected in any sample made using the two methods.

### Individual elements in aerosols from specific brands produced using the cold trap method

**Figure 4:**
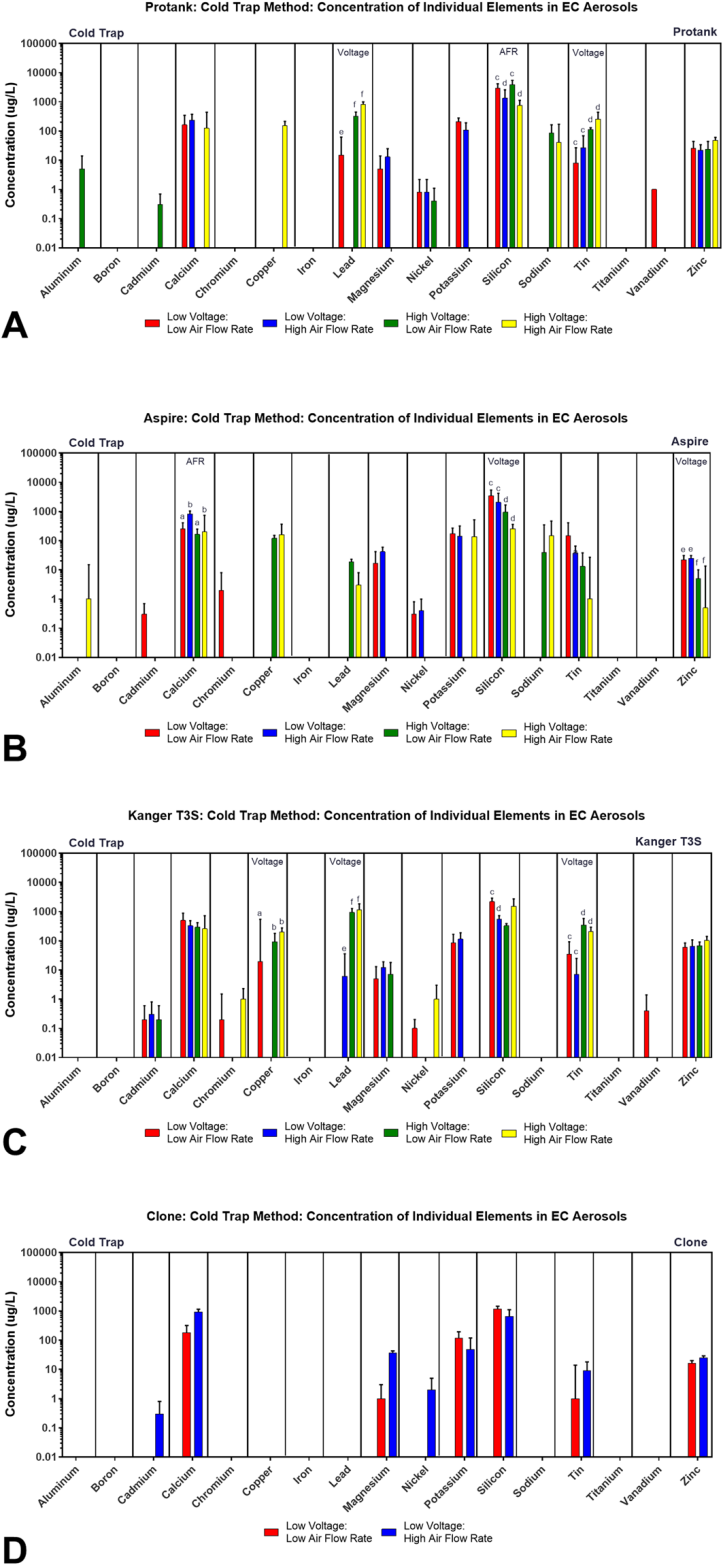
Concentrations of individual elements/metals in aerosols from four brands of tank-style EC generated using four topographic puffing parameters with the cold trap collection method. Comparison of the concentration of individual elements/metals in aerosols generated using varying voltage and air flow rate for: (**A**) Kangertech Protank, (**B**) Aspire Nautilus, (**C**) Kanger T3S, (**D**) Clone. All concentrations are the based on three independent experiments. Data samples with an “a” are significantly different from samples with “b” (p < 0.05), “c” are significantly different than “d” (p < 0.01), samples with an “e” are significantly different from samples with an “f” (p < 0.001).

Data for each element are plotted by brand for aerosols produced using the cold trap method using a combination of two different voltages and two air flow rates (Fig. 4; Table 1). The data are remarkable in that for most elements, the presence or absence of an element and its concentration were similar across all brands, irrespective of the topographic parameters. For example, zinc was present in most aerosols from all brands at about 5–102 ug/L, and its concentration was not significantly affected by topographic parameters (Fig. 4, Supplemental Tables 2–5). Elements present in the aerosols of all brands included calcium, magnesium, potassium, nickel, silicon, tin, and zinc. Some elements (e.g., aluminum, cadmium, chromium, and vanadium) were infrequently observed, and when present, their concentrations were relatively low. Some elements appeared in aerosols infrequently at low voltages, but frequently at high voltages. This group included copper, lead, and sodium. The Clone was distinct from the other three brands in having fewer different elements in its aerosols. Those elements that were present in the Clone aerosols were generally similar in concentrations to aerosols from other products, except for nickel which was higher in the Clone.

### Individual elements in aerosols produced with specific clearomizers/tanks using the impinger method

**Figure 5:**
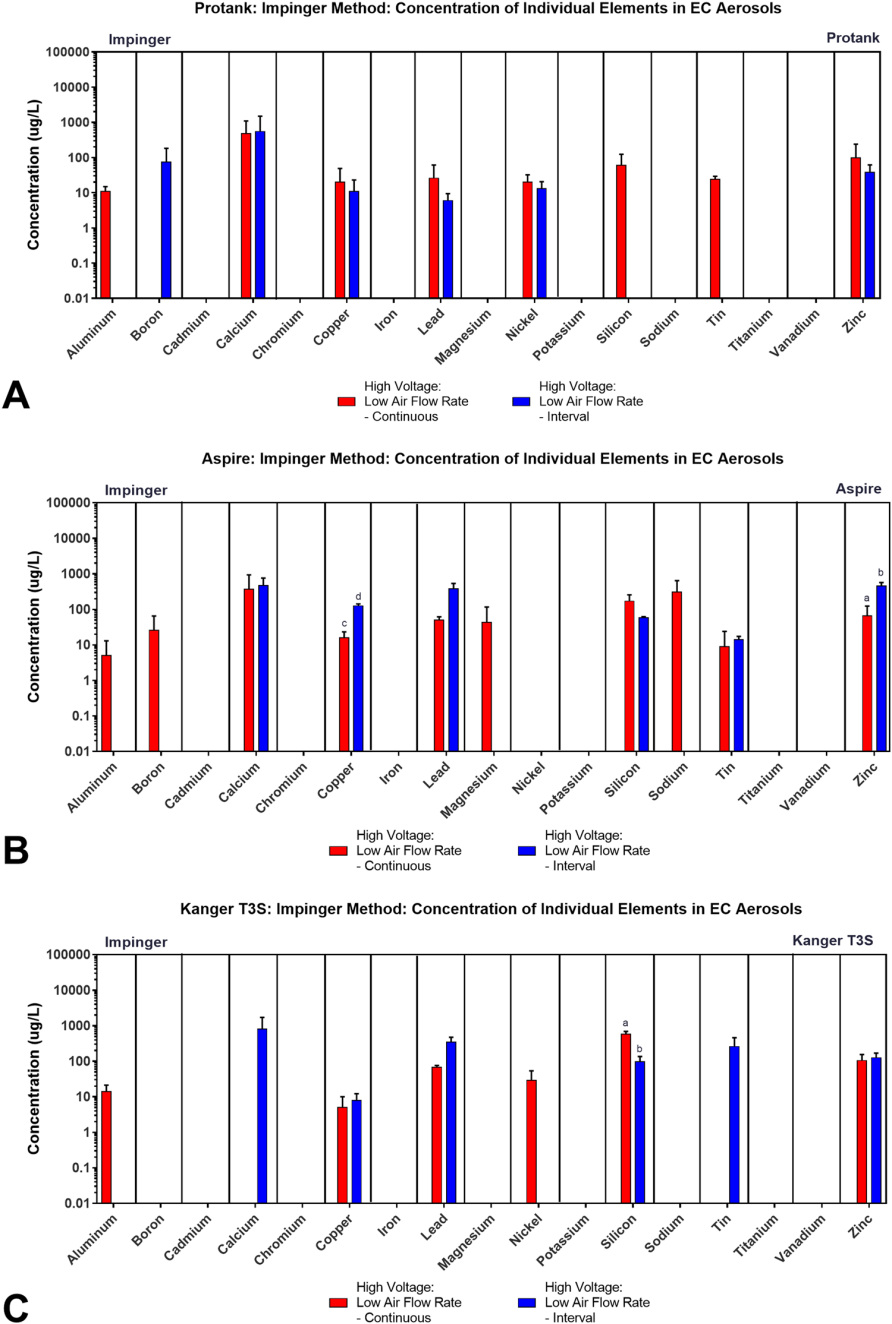
Concentrations of individual elements/metals in aerosols from three brands of tank-style EC generated using two topographic puffing parameters with the impinger collection method. Comparison of the concentration of individual elements/metals in the aerosol of classic reservoir tanks when the aerosol was generated using continuous puffing and puffs collected in intervals for: (**A**) Kangertech Protank, (**B**) Aspire Nautilus, and (**C**) Kanger T3S. All concentrations are the means of three independent experiments. Data samples with an “a” are significantly different from samples with “b” (p < 0.05), “c” are significantly different than “d” (p < 0.01).

Elements were next compared by brand for impinger-generated aerosols using high voltage, a single air flow rate (as described above, Table 1), and two puffing protocols (continuous and interval puffing). Nineteen elements were quantified in the aerosol of the six brands of tank-style EC, and the means and standard errors were plotted for the clearomizer/tank products (Fig. 5, Supplemental Tables 2–4). Of the 19 elements, six (cadmium, cobalt, potassium, silver, titanium, vanadium) were not detected in any of the aerosols (Supplemental Tables 2–4). The remaining 13 elements/metals were either detected in one or both puffing protocols (Fig. 5, Supplemental Tables 2–4).

In general, if an element was detected in the continuous protocol, it was also detected in the interval protocol (Fig. 5). Copper, lead, and zinc were present in both puffing protocols in all three brands. Some elements were only detected in one or two brands using the continuous protocol, such as aluminum, boron, magnesium, nickel, silicon, sodium, and tin. This may have occurred because the continuous protocol was done first and available elements may have been fully transferred to the aerosol before the interval protocol was used. Certain elements, such as calcium, were usually higher in concentration in the interval group than in the continuous protocol group. For Aspire and Kanger T3S, copper, lead, and zinc were all higher in the interval puffing samples (Fig. 5).

### Individual elements in aerosols produced with RDA and sub-ohm tanks using the impinger method

**Figure 6:**
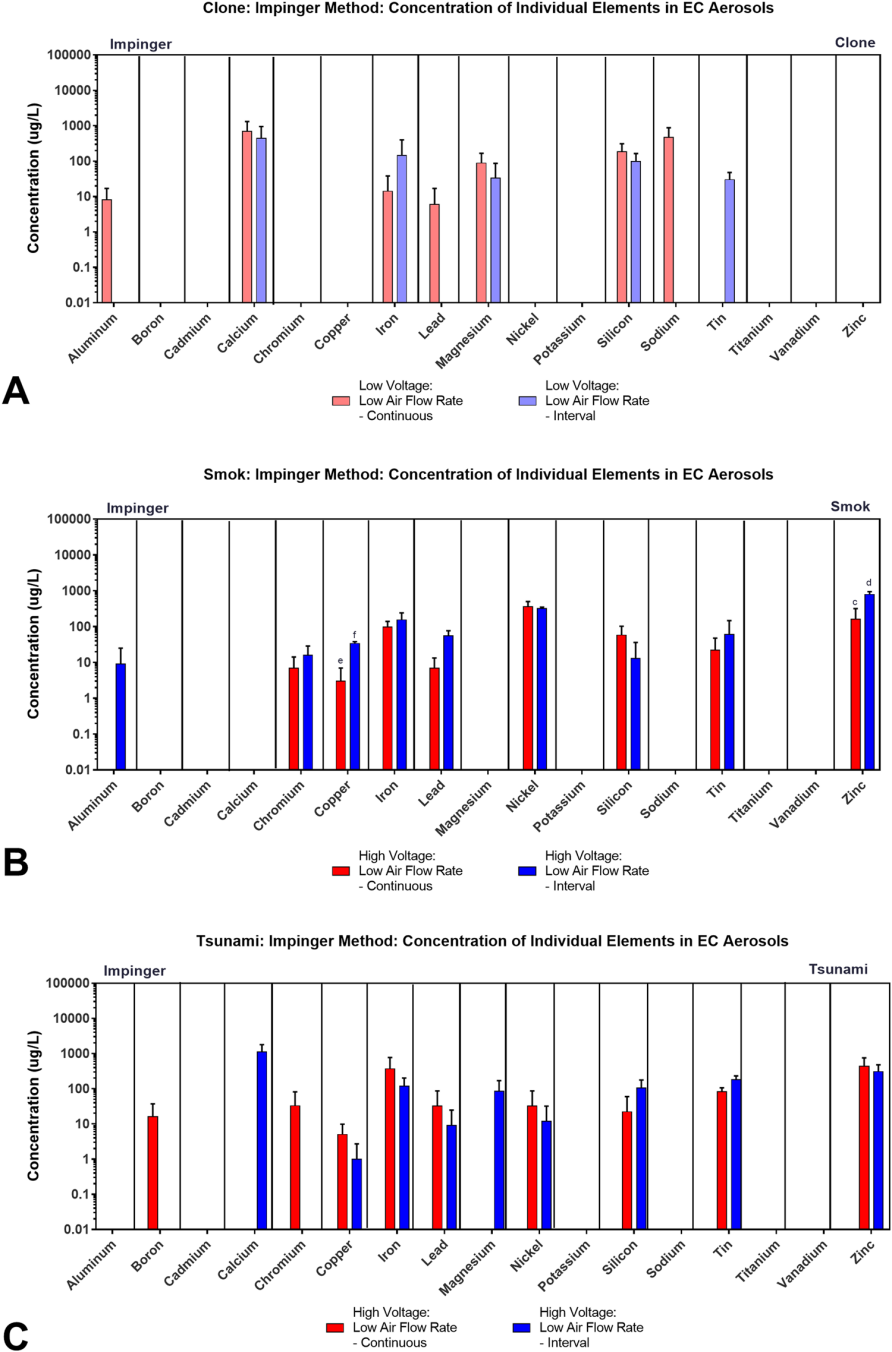
Concentrations of individual elements/metals in aerosols from three brands of sub-ohm and RDA tank-style EC generated using two topographic puffing parameters with the impinger collection method. The concentration of individual elements/metals in the aerosol of sub-ohm and RDA tanks when the aerosol was generated using continuous and interval puffing for: (**A**) Clone, (**B**) Smok, and (**C**) Tsunami 2.4. All concentrations are the based on three independent experiments. Data samples with a “c” are significantly different than “d” (p < 0.01), samples with an “e” are significantly different from samples with an “f” (p < 0.001).

Elements were next compared by brand for RDA and Sub-ohm tank-style ECs for impinger-generated aerosols using high voltage, a single air flow rate and two puffing protocols (continuous and interval puffing) (Fig. 6, Table Supplemental Tables 5–7). Of the 19 elements, six (cadmium, cobalt, potassium, silver, titanium, vanadium) were not detected in any of the aerosols. The remaining 13 elements were detected, in general, in both the continuous and interval protocols, in most cases in similar concentrations (Fig. 6). Depending on the brands, some elements were higher in the interval than in the continuous puffing (Fig. 6). In general, the results for Tsunami and Smok were similar, while Clone had fewer different elements. Iron and silicon were found with both protocols in all three brands (Fig. 6, Supplemental Tables 5–7). In the RDA brands (Clone and Tsunami), four elements (boron, chromium, lead, sodium) were detected when puffed with the continuous protocol (Fig. 6), while three elements (calcium, magnesium, tin) were detected with interval puffing. Aluminum was detected in the Clone using continuous puffing, while in Smok it was detected with interval puffing (Fig. 6A,B). In general, the sub-ohm styles had more elements that the classic tanks.

## Discussion

### Over view

This is the first study using controlled laboratory conditions that compares: (1) the contribution of collection vessels to elements/metals in the analyte, (2) the elements/metals in aerosols produced by different tank-style EC, (3) the efficiencies of two methods of aerosol collection, and (4) different topographies for aerosol production. Acid presoaking of the glassware used for aerosol collection was necessary to remove elements that leach from glass and could add to the concentrations of elements measured in aerosols. Of 19 elements/metals screened, three (cobalt, silver, titanium) were not detected in any samples. It is likely that some of the elements in tank aerosols, such as aluminum, calcium, chromium, copper, iron, lead, magnesium, nickel, silicon, tin, and zinc, were from components in the atomizing units. The total concentrations of elements/metals in aerosols collected with the cold trap method (1,226 to 6,767 µg/L) was higher than that for the impinger method (43 to 3,138 µg/L). The impinger method had the advantages of being faster to perform, collecting some elements not found with the cold trap method, and avoiding surfaces, such as tubing, that could contribute elements to the aerosols leading to an overestimation of total concentrations. For total concentrations of individual elements averaged for all brands, occasional differences were observed with different topographies, but in general concentrations were similar across topographies, as well as with the two methods of collection. The concentrations of some elements, such as lead, were significantly higher in aerosols produced at high voltages. When comparing individual elements across brands, results were again remarkably similar. For example, with the exception of one brand, zinc appeared in all aerosols irrespective of topography. Lead appeared in all aerosols, except those made with Clone, which had a simple atomizer and overall fewer elements in its aerosols. These data provide a useful benchmark for element/metal concentrations in aerosols made from a range of tank-style EC used with different topography parameters.

### Leaching of elements from glassware

Other EC reports have not addressed leaching from glassware as a possible source of contaminants that affect concentrations of elements in EC aerosols, but leaching from filters used in cigarette smoke analysis has been reported^41^. Our data demonstrate the importance of establishing that elements do not leach into the aerosol solution from surfaces used in collection and taking this into account when computing final concentrations of elements. Acid corrosion can occur in glass by creating pores in the silica scaffold thereby leaching the alkali components of the glass and bringing them into solutions^42^, which could explain why there was some potassium in the impinger acid solutions even after 5 days of soaking. In addition to pretreating glassware, all plasticware should be pretreated with acid to seal it^43^. It is also important to minimize the amount of time a sample is stored before analysis, as elements could leach during storage and contribute contaminants to the aerosol solutions.

### Methods of aerosol collection

There is currently no standard method for EC aerosol collection for metal analysis^4^. Therefore, labs have used various methods, such as glass washing bottles with methanol in dry ice, quartz filters, and condensation using pipette tips and narrow tubing^31,44,45^; however, these have been used without examining how the method affects the element concentration in aerosols. As our study shows, element concentrations can vary with the method of collection. The total concentration of elements in the cold trap high voltage low air flow rate group was about 3.5 times higher than the continuous impinger method. This could be due to: (1) leaching of elements in the cold trap method from the peristaltic pump tubing or plastic storage tubes, which were not pretreated in acid, (2) more efficient collection of all aerosol with the cold trap method, (3) the longer time (6 minutes) between puffs with the cold trap may have enabled more complete collection of the aerosol, and (4) the cold trap was a better method of collection for silicon and calcium, which contributed to the higher total concentration. It is also important to note that the cold trap method was better at collecting the alkali (sodium and potassium) and alkaline earth metals (magnesium and calcium) and metalloids (silicon, boron), but not as efficient as the impinger method at collecting the transition (heavy) metals (chromium, iron, nickel, zinc, and copper). Although we do not know the reason for these different efficiencies, these data clearly show that the method of collection can affect concentrations and that not all elements were affected in the same way. The use of two different methods provides insight into ranges of elements in EC aerosols and may help understand differences in values reported in prior literature.

Aluminum, boron, iron and nickel were present in higher concentrations in aerosols collected with the impinger method than with the cold trap. This may be due to better mixing of the aerosols with the larger volume of solvent in the impinger or loss of some elements in plastic storage tubes that were not acid sealed in the cold trap method. We recommend the use of the impinger method in conjunction with presoaking the impingers in nitric acid until leaching stops and storing aerosols in acid pre-sealed tubes with analysis as soon as possible after collection.

### Effects of topography

Some elements were only present in samples prepared using specific topographic parameters. With the cold trap method, aluminum, copper, and lead were generally detected in samples prepared using high voltage, suggesting that the EC must heat high enough to drive these elements/metals into the aerosol. These same three elements were detected in all impinger samples, which were all prepared using high voltages. In cases in which an element was present only in aerosols created at low voltage (e.g., low air flow rate for aluminum with impinger method - Fig. 5B) or only in aerosols created with continues puffing (e.g., aluminum and sodium Fig. 3B), it is possible that the element was part of a coating that was released during the initial use of the EC and no additional aluminum was available for aerosolization with the subsequent topographies.

The impinger method results are generally similar for each element within a brand. Aerosols created with the continuous puffing protocol usually contained more elements than aerosols made with the interval method, while the interval puffing protocol produced aerosols that generally had somewhat higher concentrations of individual elements (e.g., lead) than those produced by the continuous protocol (Fig. 3B). Although the reason for the higher concentration with interval puffing is not known, the cycling of the filament through hot and cold temperatures could make it more friable and prone to release more elements. The interval puffing protocol is more similar to the way a consumer would use the product and probably better represents actual user exposure.

### Dominant elements

Those elements/metals that were dominant in the aerosols, i.e. appeared in all or almost all samples (aluminum, calcium, chromium, copper, iron, lead, magnesium, nickel, silicon, sodium, tin and zinc) have been reported previously in the atomizing units of cartomizer and disposable EC products^28–30,46^, and it is likely that they originated in the atomizers. The Clone had the fewest metal parts and the fewest types of metal in the atomizer, and also had the fewest number of elements in its aerosol. The elements that are present in aerosols from the Clone are in similar concentrations to those in other products. These data suggest that reducing metal components in atomizers will decrease metals in aerosols, in support of our prior study^29^. It is also possible that some elements/metals in aerosols originated in the e-fluid, as one prior study reported^26^. Although not included in the current study, we have unpublished data on elements in a spectrum of EC fluids. Only sodium was high enough in some fluids we used to affect the data in this study. In fact, the difference seen in sodium in Fig. 3B is likely due, at least in part, to a high level of sodium in the refill fluid used for the continuous but not the interval puffing.

*Source of elements/metals in aerosols:* The concentration of elements/metals in e-fluids is higher after an EC has been used^26^, supporting the idea that metals in aerosols come from heated components in the atomizers. Some elements, such as lead, potassium, sodium, and zinc, have relatively low melting points (321 °C, 64 °C, 98 °C, 420 °C respectively) that would facilitate their transfer into aerosols when ECs heat up to 320 °C (Supplemental Table 1)^25^. Zinc was commonly found in aerosols, suggesting these devices heat up to over 320 °C. The atomizing units of the ECs used in this study did not contain lead^46^ nor did the refill fluids. Thus the source of the lead has not yet been determined for these products, but could be the glass or metals components of the tank/reservoir.

### Number and concentration of elements are affected by model and method

The number of elements in the aerosols varied with method of collection and also with the model of the EC. The interval method produced a significantly higher concentration of copper and zinc in the aerosols from Aspire and Smok products than the continuous method. This is important since it more closely resembles how an EC would actually be used. The higher concentrations of chromium, copper, and iron in the impinger aerosols of Smok and Tsunami suggest that the sub-ohm batteries and newer tanks deliver more metals into the aerosols than the older models of tank-style EC.

### Comparison to prior data

The range of total concentration of elements/metals in the aerosols of tank-style EC in the current study (374 to 3,028 µg/L) was similar to that found previously in disposable EC (973 to 2,296 µg/L)^30^. A group recently screened 15 elements in the aerosols from different brands of tank-style EC using a condensation method of collection^26^. For the subset of eight elements (aluminum, chromium, copper, iron, lead, nickel, tin, zinc) that were present in the current and preceding studies, the total median concentrations were 670.04 µg/L (tanks – condensation collection)^26^ 101.172 µg/L (disposable -cold trap collection)^30^, 161.44 µg/L (tanks - cold trap collection- current study), and 441.30 µg/L (tanks - impinger collection- current study). For this subset of elements, the median concentrations of the impinger (current study) and the Olmedo *et al*. 2018 study are in reasonable agreement. However, the subset medians for both cold trap methods are lower than that for the tank condensation and impinger collection methods. These differences could be due to less efficient collection of certain elements using the cold trap method, lower concentrations of elements in the aerosols produced by the lower voltage disposable models, the use of different EC models/brands in each study, or a combination of these factors. The importance of voltage/power is shown by the observation that some elements (aluminum, boron, copper, iron, lead, sodium) were only produced at the higher voltage.

### Comparison to cigarette smoke

The total concentration of elements/metals in the aerosol of tank-style EC (226–6,767 µg/L) was higher than that found in cigarette smoke prepared using the International Organization for Standardization (ISO) (2,690 µg/L), Health Canadian Standard (HCS) protocols (1,103 µg/L)^30^. Of the 19 elements screened in this study, four (boron, iron, silver, titanium) were present in cigarette smoke and not in EC aerosol prepared using the cold trap method. However, some elements (aluminum, cadmium) were present in EC aerosol and not in cigarette smoke. Four elements (copper, lead, nickel, zinc) were present in both EC aerosol and cigarettes smoke, and both lead (407 µg/L) and zinc (36 µg/L) were found in higher concentrations in EC aerosol than in cigarette smoke (ISO – 0.126 µg/L, HCS – 1.252 µg/L)^30^. The concentration of copper and nickel in cigarette smoke was within the range in EC aerosol (nickel: ISO - 0.655 µg/L, HCS – 2.769 µg/L, EC - 0.074–2.3 µg/L, copper: ISO – 80 µg/L, HCS – 170 µg/L, EC - 19–200 µg/L)^30^. Other studies have reported that individual metals in cigarette smoke prepared using the HCS usually had a higher concentration of metals than samples prepared using the ISO protocol^41,47–49^. For example, the concentrations in Marlboro Red cigarette aerosols were two to three times higher in samples prepared using the HCS^47^.

### Potential health effects of EC elements/metals

The potential health effects of elements and metals in EC aerosols have recently been reviewed^34,50,51^. Chromium, lead, and nickel are of particular concern as they are known carcinogens^32^. Prolonged exposure to chromium from EC aerosol could cause gastrointestinal effects, nasal and lung cancer, respiratory irritation, and lung function impairment^34,52–54^. Tank-style EC deliver higher concentrations of nickel than previous EC models^28–30^. Nickel inhalation can cause lung disease, damage to the nasal cavity, lung irritation, lung inflammation, hyperplasia in pulmonary cells, and fibrosis^53,55,56^. Prolonged exposure to lead, which has been found in varying concentrations in all styles of EC, could produce vomiting, diarrhea, cardiovascular effects, and lung cancer^34^. Olmedo *et al*. 2018 also reported that concentrations of chromium, lead, and nickel are high enough in EC aerosols to be a health risk^26^. Likewise the concentrations of some elements (chromium, copper, lead, nickel, zinc) reported in our study exceed the proposed Occupational Safety and Health Administration, permissible exposure limit (OSHA PEL)^34^. For example, the OSHA PEL for chromium is 5 × 10^3^ ng/m^3 ^^34^, and the concentration of chromium found in one brand of tank-style EC (Tsunami 2.4) was 3.3 × 10^7^ ng/m^3^, which is much higher than the OSHA PEL. Because, OSHA values are for occupational not recreational exposure, our values may underestimate potential harm to EC users.

### Speciation

Since most methods of measuring metals in aerosol samples only report concentration and not speciation, it is not yet known if the species of chromium, nickel and lead would be harmful. For example, chromium (III) is an essential nutrient in the human diet and not readily absorbed by cells, but its reduction to Cr(VI) could cause oxidative stress, DNA adducts, DNA-protein crosslinks, and damage to lipid bilayers in cells^57,58^. In addition, exposure to Cr(VI) is a respiratory irritant and could lead to nasal, sinus, and lung cancer^54^.

## Conclusions

Tank-style EC have evolved to provide larger puffs, store larger amounts of refill fluid, and allow for more customizability by the consumer. These changes enable operation of products at higher voltage/power, which correlates with increased concentrations of several elements/metals (including lead, nickel, iron, copper) in their aerosols. Acid pre-cleaning of collection vessels was important to remove elements that are readily leached by nitric acid. Results varied somewhat with the two collection methods. The impinger method had the advantages of being more rapid to perform, able to collect elements (including heavy metals) that were not collected efficiently with the cold trap, and able to collect aerosols without tubing, which can trap aerosol elements or release contaminating elements. However, the impinger method did require 5 days of pre-cleaning with acid to remove leachable elements. In some cases, topography did affect the concentration of elements that were transferred to the aerosol, with more being transferred when ECs were puffed using higher voltages/power. The total concentration of elements in EC aerosols has increased with the evolution from cig-a-like to the tank-style models, which generate higher power. The concentration of individual elements was similar across collection methods, topography, and brand, with the exception of aluminum, copper, iron, lead, nickel, sodium, and tin, which were higher in concentrations in samples generated using high voltages/power. Most of the elements/metals in the aerosols likely originated from the atomizing unit. These data will be helpful to regulatory agencies, healthcare providers, and consumers, and will help understand the health effects associated with the use of tank-style EC and the concentrations of elements/metals they deliver.

## Supplementary information

Supplementary Materials
