# Supplementary material for: Effects of Model, Method of Collection, and Topography on Chemical Elements and Metals in the Aerosol of Tank-Style Electronic Cigarettes
Source: Sci Rep. 2019 Sep 27;9:13969. doi: 10.1038/s41598-019-50441-4 (PMC6765047; doi:10.1038/s41598-019-50441-4)
Supplement: Supplementary file 1 — Supplementary Materials [file 41598_2019_50441_MOESM1_ESM.docx]

Effects of Model, Method of Collection, and Topography on Chemical Elements and Metals in the Aerosol of Tank-Style Electronic Cigarettes

Monique Williams^1^, Jun Li^2^, and Prue Talbot^1*^

^1^Department of Cell Biology and Neuroscience, University of California, Riverside, Riverside, California, USA

^2^Department of Statistics, University of California, Riverside, Riverside, California, USA

^*^Corresponding author

Email: [talbot@ucr.edu](mailto:talbot@ucr.edu)

Running title: Elements and Metals in Tank Mod Style EC

**Supplemental Material**

**ICP-OES Analysis of metals, running conditions, and quality control**

Water and reagents: For ICP-OES, Milli-Q System water (Millipore, USA) at 18.2 mOhm of resistance was used to make the nitric acid solutions. Nitric acid was purchased from Macron Chemicals (Avanter Performance Materials, Inc, Center Valley, PA) and was AR Select (ACS) for Trace Element Analysis. Hydrochloric acid (cold trap samples) was purchased from Fisher Scientific (Fair Lawn, NJ) and was Certified ACS PLUS.

Analysis of metals: An ICP OES Perkin –Elmer Optima 7300 DV (Perkin Elmer USA) with an autosampler, a Perkin-Elmer Nebulizer (N0777036 REV A, Cyclonic spray chamber Optima 5300DV, Quartz 7mm baffle drain line) and a segmented array detector charge coupled device detector (SCD) was used for all analyses. The ICP-OES was calibrated daily using Perkin-Elmer Multi-element calibration standards Plus #2, #3, #4, and #5. Quality control checks on calibration were then run using NIST standard reference materials by Ultra Scientific (trace metal sample, QCI-700A, North Kingstown, RI). Running conditions were plasma flow = 15 L/min, auxiliary flow 0.2 L/min, nebulizer flow of 0.75 L/min, radio frequency power 1450 W, sample flow rate= 0.80 mL/min, and a read delay time of 12 sec. For an internal standard, yttrium at 2.5 ppm was run in line with sample introduction into the nebulizer. Distilled deionized water with 1% nitric acid was run as the blank. Each sample was run in triplicate. When interference was observed for any element, additional peaks were monitored to identify the best wavelength for quantification. The concentrations of each element in the blank were subtracted from the measured concentrations in each sample. Samples of room air made the same way as the EC aerosol samples were run with each batch of samples, and room air values were subtracted from measured concentrations of each element in the aerosols.

In addition to the quality controls and standards used to calibrate the instrument, additional standard solutions were prepared at a 0.25 and 1.0 ppm of 9 analytes (aluminum, chromium, copper, lead, nickel, selenium, silicon, tin, zinc) (Inorganic Ventures, Christiansburg, VA) in 2% nitric acid (Avanter Performance Materials, Inc, Center Valley, PA) and 18.2 mOhm deionized water. These prepared standard concentrations were used to assess the instruments ability to measure concentrations accurately.

Supplemental Table 1 Elemental Class, Limits of Detection, Melting Point, Boiling Point for Elements in EC Aerosols

| **Element** | **Elemental Class** | **Limit of Detection (mg/L)** | **Melting Point**  (°C) | **Boiling Point**  (°C) |
| --- | --- | --- | --- | --- |
| Aluminum | Post-Transition | 0.0043 | 660 | 2519 |
| Boron | Metalloids | 0.0028 | 2075 | 4000 |
| Cadmium | Transition | 0.00069 | 321 | 767 |
| Calcium | Alkaline Earth | 0.0026 | 842 | 1484 |
| Chromium | Transition | 0.0020 | 1907 | 2671 |
| Cobalt | Transition | 0.0010 | 1495 | 2927 |
| Copper | Transition | 0.0024 | 1084 | 2562 |
| Iron | Transition | 0.00066 | 1538 | 2861 |
| Lead | Post-Transition | 0.0067 | 327 | 1749 |
| Magnesium | Alkaline Earth | 0.00047 | 650 | 1090 |
| Nickel | Transition | 0.00081 | 1455 | 2913 |
| Potassium | Alkali | 0.0017 | 64 | 759 |
| Silicon | Metalloids | 0.0027 | 1414 | 2900 |
| Silver | Transition | 0.0020 | 962 | 2162 |
| Sodium | Alkali | 0.0111 | 98 | 883 |
| Tin | Post-Transition | 0.0040 | 232 | 2602 |
| Titanium | Transition | 0.00035 | 1668 | 3287 |
| Vanadium | Transition | 0.0011 | 1910 | 3407 |
| Zinc | Transition | 0.00050 | 420 | 907 |

Supplemental Table 2: Total and individual concentrations of elements in Kangertech Protank reservoir tank style EC aerosol in µg/L

| **Ego C-Twist – Kangertech Protank** | | | | | | |
| --- | --- | --- | --- | --- | --- | --- |
| **Element** | **Cold Trap** | | | | **Impinger** | |
|  | **Continuous** | **Continuous** | **Continuous** | **Continuous** | **Continuous** | **Interval** |
|  | **LV: LAFR** | **LV: HAFR** | **HV: LAFR** | **HV: HAFR** | **HV: LAFR** | **HV: LAFR** |
| **Aluminum** |  |  | 5 ± 9 |  | 11 ± 4 |  |
| **Boron** |  |  |  |  |  | 75 ± 108 |
| **Cadmium** |  |  | 0.3 ± 0.4 |  |  |  |
| **Calcium** | 162 ± 184 | 235 ± 139 |  | 124 ± 315 | 475 ± 615 | 549 ± 941 |
| **Chromium** |  |  |  |  |  |  |
| **Copper** |  |  |  | 150 ± 64 | 20 ± 29 | 11 ± 12 |
| **Iron** |  |  |  |  |  |  |
| **Lead** | 15 ± 47 |  | 320 ± 127 | 827 ± 167 | 26 ± 35 | 6 ± 3 |
| **Magnesium** | 5 ± 9 | 13 ± 12 |  |  |  |  |
| **Nickel** | 0.8 ± 1.4 | 0.8 ± 1.4 | 0.4 ± 0.7 |  | 20 ± 12 | 13 ± 8 |
| **Potassium** | 213 ± 67 | 109 ± 83 |  |  |  |  |
| **Silicon** | 2887 ± 1278 | 1328 ± 1247 | 3826 ± 1600 | 742 ± 390 | 60 ± 64 |  |
| **Sodium** |  |  | 87 ± 76 | 41 ± 130 |  |  |
| **Tin** | 8 ± 19 | 27 ± 42 | 112 ± 18 | 254 ± 187 | 24 ± 5 |  |
| **Titanium** |  |  |  |  |  |  |
| **Vanadium** | 1 ± 0 |  |  |  |  |  |
| **Zinc** | 26 ± 18 | 22 ± 12 | 24 ± 20 | 48 ± 13 | 99 ± 141 | 38 ± 24 |
| **Total Conc – No Al, Cr, Pb,Sn** | **3347** ± 1269 | **1785** ± 1437 | **4382** ± 1479 | **2323** ± 525 |  |  |
| **Total Conc – No B, Mg, K, Ti** |  |  |  |  | **735** ± 902 | **617** ± 944 |

Supplemental Table 3: Total and individual concentrations of elements in Aspire Nautilus reservoir tank style EC aerosol in µg/L

| **Ego C-Twist – Aspire Nautilus** | | | | | | |
| --- | --- | --- | --- | --- | --- | --- |
| **Element** | **Cold Trap** | | | | **Impinger** | |
|  | **Continuous** | **Continuous** | **Continuous** | **Continuous** | **Continuous** | **Interval** |
|  | **LV: LAFR** | **LV: HAFR** | **HV: LAFR** | **HV: HAFR** | **HV: LAFR** | **HV: LAFR** |
| **Aluminum** |  |  |  | 1 ± 14 | 5 ± 8 |  |
| **Boron** |  |  |  |  | 26 ± 39 |  |
| **Cadmium** | 0.3 ± 0.4 |  |  |  |  |  |
| **Calcium** | 251 ± 160 | 832 ± 208 | 164 ± 84 | 200 ± 540 | 367 ± 566 | 468 ± 292 |
| **Chromium** | 2 ± 6 |  |  |  |  |  |
| **Copper** |  |  | 121 ± 31 | 162 ± 206 | 16 ± 7 | 126 ± 17 |
| **Iron** |  |  |  |  |  |  |
| **Lead** |  |  | 19 ± 4 | 3 ± 5 | 50 ± 12 | 381 ± 157 |
| **Magnesium** | 17 ± 25 | 42 ± 18 |  |  | 43 ± 74 |  |
| **Nickel** | 0.3 ± 0.5 | 0.4 ± 0.6 |  |  |  |  |
| **Potassium** | 175 ± 95 | 141 ± 179 |  | 139 ± 379 |  |  |
| **Silicon** | 3501 ± 1940 | 2037 ± 2166 | 956 ± 724 | 254 ± 106 | 171 ± 86 | 58 ± 4 |
| **Sodium** |  |  | 39 ± 309 | 150 ± 318 | 311 ± 332 |  |
| **Tin** | 147 ± 264 | 37 ± 28 | 13 ± 25 | 1 ± 26 | 9 ± 15 | 14 ± 3 |
| **Titanium** |  |  |  |  |  |  |
| **Vanadium** |  |  |  |  |  |  |
| **Zinc** | 22 ± 9 | 25 ± 6 | 5 ± 5 | 0.5 ± 13 | 66 ± 58 | 452 ± 119 |
| **Total Conc – No Al, Cr, Pb, Sn** | **4154** ± 2266 | **3157** ± 2183 | **1510** ± 795 | **1259** ± 904 |  |  |
| **Total Conc – No B, Mg, K, Ti** |  |  |  |  | **995** ± 984 | **1499** ± 260 |

Supplemental Table 4: Total and individual concentrations of elements in Kanger T3S reservoir tank style EC aerosol in µg/L

| **iTaste MVP 2.0 – Kanger T3S** | | | | | | |
| --- | --- | --- | --- | --- | --- | --- |
| **Element** | **Cold Trap** | | | | **Impinger** | |
|  | **Continuous** | **Continuous** | **Continuous** | **Continuous** | **Continuous** | **Interval** |
|  | **LV: LAFR** | **LV: HAFR** | **HV: LAFR** | **HV: HAFR** | **HV: LAFR** | **HV: LAFR** |
| **Aluminum** |  |  |  |  | 14 ± 7 |  |
| **Boron** |  |  |  |  |  |  |
| **Cadmium** | 0.2 ± 0.4 | 0.3 ± 0.5 | 0.2 ± 0.4 |  |  |  |
| **Calcium** | 499 ± 383 | 328 ± 160 | 292 ± 127 | 257 ± 465 |  | 808 ± 911 |
| **Chromium** | 0.2 ± 1.3 |  |  | 1 ± 1.3 |  |  |
| **Copper** | 19 ± 524 |  | 92 ± 88 | 201 ± 74 | 5 ± 5 | 8 ± 4 |
| **Iron** |  |  |  |  |  |  |
| **Lead** |  | 6 ± 30 | 937 ± 329 | 1133 ± 702 | 68 ± 8 | 348 ± 128 |
| **Magnesium** | 5 ± 8 | 12 ± 7 | 7 ± 11 |  |  |  |
| **Nickel** | 0.1 ± 0.1 |  |  | 1 ± 2 | 29 ± 25 |  |
| **Potassium** | 85 ± 81 | 114 ± 74 |  |  |  |  |
| **Silicon** | 2198 ± 705 | 555 ± 173 | 327 ± 59 | 1505 ± 1211 | 578 ± 112 | 96 ± 40 |
| **Sodium** |  |  |  |  |  |  |
| **Tin** | 35 ± 57 | 7 ± 18 | 345 ± 238 | 208 ± 84 |  | 260 ± 198 |
| **Titanium** |  |  |  |  |  |  |
| **Vanadium** | 0.4 ± 1 |  |  |  |  |  |
| **Zinc** | 60 ± 24 | 64 ± 43 | 67 ± 24 | 102 ± 38 | 105 ± 51 | 126 ± 43 |
| **Total Conc – No Al, Cr, Pb, Sn** | **3107** ± 379 | **1136** ± 496 | **2134** ± 784 | **3522** ± 979 |  |  |
| **Total Conc – No B, Mg, K, Ti** |  |  |  |  | **799** ± 135 | **1646** ± 759 |

Supplemental Table 5: Total and individual concentrations of elements in Clone RDA style EC aerosol in µg/L

| **Nemesis - Clone** | | | | |
| --- | --- | --- | --- | --- |
| **Element** | **Cold Trap** | | **Impinger** | |
|  | **Continuous** | **Continuous** | **Continuous** | **Interval** |
|  | **LV: LAFR** | **LV: HAFR** | **HV: LAFR** | **HV: LAFR** |
| **Aluminum** |  |  | 8 ± 9 |  |
| **Boron** |  |  |  |  |
| **Cadmium** |  | 0.3 ± 0.5 |  |  |
| **Calcium** | 182 ± 136 | 925 ± 223 | 697 ± 614 | 440 ± 508 |
| **Chromium** |  |  |  |  |
| **Copper** |  |  |  |  |
| **Iron** |  |  | 14 ± 24 | 145 ± 251 |
| **Lead** |  |  | 6 ± 11 |  |
| **Magnesium** | 1 ± 2 | 36 ± 7 | 88 ± 78 | 33 ± 53 |
| **Nickel** |  | 2 ± 3 |  |  |
| **Potassium** | 118 ± 76 | 48 ± 72 |  |  |
| **Silicon** | 1164 ± 298 | 644 ± 459 | 186 ± 123 | 98 ± 65 |
| **Sodium** |  |  | 470 ± 407 |  |
| **Tin** | 1 ± 13 | 9 ± 9 |  | 30 ± 18 |
| **Titanium** |  |  |  |  |
| **Vanadium** |  |  |  |  |
| **Zinc** | 16 ± 4 | 25 ± 4 |  |  |
| **Total Conc – No Al, Cr, Pb, Sn** | **1504** ± 505 | **1729** ± 670 |  |  |
| **Total Conc – No B, Mg, K, Ti** |  |  | **1381** ± 1168 | **712** ± 421 |

Supplemental Table 6: Total and individual concentrations of elements in Smok sub-ohm tank style EC aerosol in µg/L

| **Smok Alien - Smok** | | |
| --- | --- | --- |
| **Element** | **Impinger** | |
|  | **Continuous** | **Interval** |
|  | **HV: LAFR** | **HV: LAFR** |
| **Aluminum** |  | 9 ± 16 |
| **Boron** |  |  |
| **Cadmium** |  |  |
| **Calcium** |  |  |
| **Chromium** | 7 ± 7 | 16 ± 13 |
| **Copper** | 3 ± 4 | 34 ± 4 |
| **Iron** | 98 ± 42 | 155 ± 87 |
| **Lead** | 7 ± 6 | 55 ± 22 |
| **Magnesium** |  |  |
| **Nickel** | 361 ± 138 | 318 ± 31 |
| **Potassium** |  |  |
| **Silicon** | 58 ± 45 | 13 ± 23 |
| **Sodium** |  |  |
| **Tin** | 22 ± 26 | 60 ± 87 |
| **Titanium** |  |  |
| **Vanadium** |  |  |
| **Zinc** | 162 ± 157 | 789 ± 162 |
| **Total Conc – No B, Mg, K, Ti** | **718** ± 307 | **1449** ± 94 |

Supplemental Table 7: Total and individual concentrations of elements in Tsunami 2.4 sub-ohm RDA style EC aerosol in µg/L

| **iPV6X – Tsunami 2.4** | | |
| --- | --- | --- |
| **Element** | **Impinger** | |
|  | **Continuous** | **Interval** |
|  | **HV: LAFR** | **HV: LAFR** |
| **Aluminum** |  |  |
| **Boron** | 16 ± 21 |  |
| **Cadmium** |  |  |
| **Calcium** |  | 1125 ± 666 |
| **Chromium** | 33 ± 49 |  |
| **Copper** | 5 ± 5 | 1 ± 2 |
| **Iron** | 370 ± 402 | 119 ± 83 |
| **Lead** | 32 ± 55 | 9 ± 16 |
| **Magnesium** |  | 85 ± 84 |
| **Nickel** | 32 ± 55 | 12 ± 20 |
| **Potassium** |  |  |
| **Silicon** | 22 ± 38 | 106 ± 71 |
| **Sodium** |  |  |
| **Tin** | 83 ± 23 | 182 ± 50 |
| **Titanium** |  |  |
| **Vanadium** |  |  |
| **Zinc** | 435 ± 316 | 307 ± 174 |
| **Total Conc – No B, Mg, K, Ti** | **1011** ± 930 | **1860** ± 982 |
